# Genome-wide identification and expression analysis of HSP90 gene family in *Nicotiana tabacum*

**DOI:** 10.1186/s12863-019-0738-8

**Published:** 2019-03-19

**Authors:** Zhaopeng Song, Feilong Pan, Chao Yang, Hongfang Jia, Houlong Jiang, Fan He, Najia Li, Xiaochong Lu, Hongying Zhang

**Affiliations:** 1 Key Laboratory for Cultivation of Tobacco Industry, College of Tobacco Science, Henan Agricultural University, Zhengzhou, 450002 China; 2 Chongqing Tobacco Science Research Institute, Chongqing, 400715 China

**Keywords:** Heat shock protein 90, *Nicotiana tabacum*, Genome-wide analysis, Phylogenetic analysis, Expression pattern

## Abstract

**Background:**

Heat shock proteins 90 (HSP90s) are a highly conserved protein family of cellular chaperones widely found in plants; they play a fundamental role in response to biotic and abiotic stresses. The genome-wide analysis of HSP90 gene family has been completed for some species; however, it has been rarely reported for the tobacco HSP90 genes.

**Results:**

In this study, we systematically conducted genome-wide identification and expression analysis of the tobacco HSP90 gene family, including gene structures, evolutionary relationships, chromosomal locations, conserved domains, and expression patterns. Twenty-one *NtHSP90s* were identified and classified into eleven categories (*NtHSP90–1* to *NtHSP90–11*) based on phylogenetic analysis. The conserved structures and motifs of NtHSP90 proteins in the same subfamily were highly consistent. Most NtHSP90 proteins contained the ATPase domain, which was closely related to conserved motif 2. Motif 5 was a low complexity sequence and had the function of signal peptide. At least 6 pairs of *NtHSP90* genes underwent gene duplication, which arose from segment duplication and tandem duplication events. Phylogenetic analysis showed that most species expanded according to their own species-specific approach during the evolution of HSP90s. Dynamic expression analysis indicated that some *NtHSP90* genes may play fundamental roles in regulation of abiotic stress response. The expression of *NtHSP90–4*, *NtHSP90–5*, and *NtHSP90–9* were up-regulated, while *NtHSP90–6*, and *NtHSP90–7* were not induced by ABA, drought, salt, cold and heat stresses. Among the five treatments, *NtHSP90s* were most strongly induced by heat stress, and weakly activated by ABA treatment. There was a similar response pattern of *NtHSP90s* under osmotic stress, or extreme temperature stress.

**Conclusions:**

This is the first genome-wide analysis of Hsp90 in *N. tabacum*. These results indicate that each *NtHSP90* member fulfilled distinct functions in response to various abiotic stresses.

**Electronic supplementary material:**

The online version of this article (10.1186/s12863-019-0738-8) contains supplementary material, which is available to authorized users.

## Background

Plants are often affected by a variety of strenuous stresses during growth and development, including biotic and abiotic stresses, all of which are interrelated [1]. Moreover, the main abiotic stresses such as cold, drought, salinity, freezing, high light intensity, ozone (O_3_), and heat have a critical impact on the quality and yield of plants [2–5]. Recently, with global warming, heat stress has become one of the main abiotic stresses that affect the normal growth and development of plants all over the world [6–8].

Over the course of long-term evolution, plants form regulatory mechanisms which are resistant to adverse environmental conditions. When the plants are stimulated by heat or other factors, they produce highly conserved stress proteins called heat shock proteins (HSPs) [9–11]. Many types of HSPs have been identified in almost all organisms [12]. Heat shock proteins have been classified into HSP100/ClpB family, HSP90 family, HSP70/DnaK family, chaperonin (HSP60/GroEL) family, and small heat-shock proteins (sHSP) family based on their approximate molecular weights [3, 13–15].

Heat shock protein 90 family is a widespread class of molecular chaperones in eukaryotic cytoplasm, which is highly conserved [16–18]. For example, there are seven HSP90s in *Arabidopsis*, of which AtHSP90–1, AtHSP90–2, AtHSP90–3, and AtHSP90–4 are located in the cytoplasm, and AtHSP90–5, AtHSP90–6, and AtHSP90–7 are located in the chloroplast, mitochondria and endoplasmic reticulum, respectively [19, 20]. HSP90 is an ATP-regulated dimeric chaperone mainly consisting of three highly conserved domains: the C-terminal domain of about 25 kDa that binds to the substrate, the 35 kDa intermediate domain, and the 12 kDa N-terminal domain of the ATP-binding (NTD) [21–23]. HSP90s are part of the GHKL superfamily (HSP90, histidine kinase, MutL, and gyrases) of ATPases [24]. In HSP90s, the grooves that combine with ATP are often in a closed state [25], and the N-terminal ATPase activity is low [26]. When present in the cytoplasm of eukaryotic cells, HSP90s have a charged region between the middle domain and the N-terminal domain; the charged regions of different species have different lengths [27]. It is known that the function of any protein is determined by the formation and folding into a three-dimensional structure [28]. HSP90, as a class of chaperones, is mainly involved in the formation of the spatial structure of kinase substrate, DNA repair and substrate activation, initial stress signaling, the maintenance of the spatial structure of transcription factors, etc. [29–33]. Under stress or normal conditions, the HSP90 gene family has the function of preventing the aggregation of proteins and facilitating the refolding of inactive proteins [34], which together with other chaperones present in the organism forms a mechanism that assists in protein folding [3]. When plants are stressed, the expression of stressor HSP90 is up-regulated; it interacts with non-proteinaceous substances, and repairs the deformed protein [35].

Nine and seven HSP90 genes were found in *Oryza sative* [36] and *Arabidopsis thaliana*, respectively [19]. However, the identification of the tobacco HSP90 gene family has not yet been completed. Tobacco is an important economic crop and a typical model plant. Research on the tobacco HSP90 genes is of great significance for other plants [37]. The completion of genome-wide sequencing of tobacco provides the necessary information for data mining of HSP90 at the whole genome level [38]. In this study, we performed a genome-wide survey of Sol Genomics Network databases using HSP90 protein sequences from *Arabidopsis*. Bioinformatics methods were used to analyze gene structures, evolutionary relationships, chromosomal locations, and conserved domains of the tobacco HSP90 family in detail. In addition, we studied the expression patterns of the *NtHSP90* genes under different abiotic stresses by qRT-PCR. The results are significant for the growth and development of tobacco and would provide a basis for further study of the biological functions of Hsp90 genes.

## Results

### Identification of the HSP90 gene family in *Nicotiana tabacum*

**Fig. 1:**
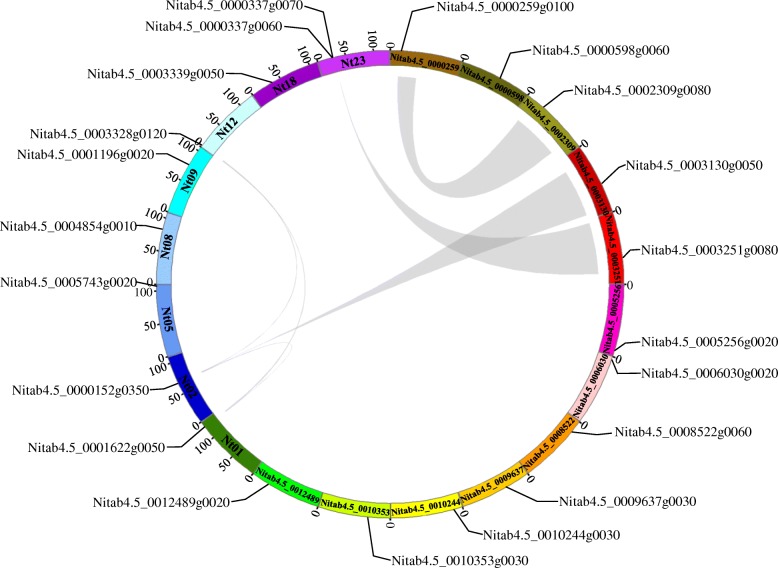
Chromosomal location of HSP90 gene members in *Nicotiana tabacum* genome. Gray lines represent gene duplications

A local BLASTP search was used to identify HSP90 members in the tobacco genome using the *Arabidopsis* HSP90 protein sequence as a query sequence. We detected 21 predicted candidate HSP90 family proteins. In tobacco, the HSP90 genes were not randomly distributed on each chromosome; there were many gene clusters on the chromosome. Nine *NtHSP90s* were mapped onto 8 chromosomes and two *NtHSP90s* were located on chromosome 23. However, 12 *NtHSP90s* could not be conclusively localized to a chromosome (Fig. 1). In addition, there were at least 6 pairs of *NtHSP90* genes that underwent gene duplication, which was possibly caused by segment duplication and tandem duplication events. Segment duplication resulted in many homologies of HSP90 genes between the chromosomes, which widened the HSP90 genome of tobacco. For example, Nitab4.5_0000152g0350 and Nitab4.5_0001622g0050 were the products of genomic segment replication.

The biophysical properties of coding HSP90s were calculated using the Expasy ProtParam tool. As shown in Table 1, the length of coding HSP90 sequences ranged from 594 to 2517 bp. The number of amino acids and biophysical properties of proteins encoded by different HSP90 genes were different, with amino acid number ranging from 197 to 838 on chromosomes. The molecular weight of different HSP90s varied greatly, and the fluctuation range was from 21,871.64 Da to 95,717.25 Da. The number of exons of the coding proteins ranged from 4 to 20. The isoelectric point (pI) of all HSP90s was acidic, indicating that the HSPs of tobacco were rich in acidic amino acids. Among them, the protein of Nitab4.5_0009637g0030 had the highest isoelectric point of 5.9430.

**Table 1:** The information of the HSP90 gene family in *Nicotiana tabacum*

Gene locus	Chromosomes	Start	End	Exon number	Gene length	CDS (bp)	Number of amino acids	Molecular mass (Da)	pI
Nitab4.5_0001622g0050	Nt01	123,900,072	123,903,771	4	3700	594	197	21,871.64	4.1058
Nitab4.5_0000152g0350	Nt02	68,363,523	68,366,651	4	3129	1416	471	53,021.11	4.5122
Nitab4.5_0005743g0020	Nt05	108,315,158	108,323,137	5	7980	837	278	31,300.40	4.3901
Nitab4.5_0004854g0010	Nt08	81,567,562	81,571,115	4	3554	1092	363	40,999.02	4.2511
Nitab4.5_0001196g0020	Nt09	75,962,866	75,971,286	19	8421	2517	838	95,717.25	4.7986
Nitab4.5_0003328g0120	Nt12	2,802,222	2,808,157	4	5935	801	266	30,326.05	4.6460
Nitab4.5_0003339g0050	Nt18	36,437,905	36,446,277	19	8373	2475	824	94,504.93	4.8689
Nitab4.5_0000337g0060	Nt23	29,167,663	29,172,699	15	5037	2463	820	94,071.92	4.6014
Nitab4.5_0000337g0070	Nt23	29,188,054	29,193,370	15	5317	2439	812	93,068.69	4.5818
Nitab4.5_0003130g0050	Nitab4.5_0003130	196,863	199,986	4	3124	1443	480	54,018.34	4.5764
Nitab4.5_0009637g0030	Nitab4.5_0009637	30,714	33,800	6	3086	1197	398	46,105.06	5.9430
Nitab4.5_0000598g0060	Nitab4.5_0000598	474,969	477,958	4	2990	1383	460	51,730.69	4.5368
Nitab4.5_0002309g0080	Nitab4.5_0002309	103,335	110,770	19	7436	2397	798	90,983.62	4.6916
Nitab4.5_0010353g0030	Nitab4.5_0010353	19,876	25,188	20	5313	2376	791	89,914.39	5.0743
Nitab4.5_0003251g0080	Nitab4.5_0003251	199,559	206,826	16	7267	2436	811	93,105.68	4.7401
Nitab4.5_0008522g0060	Nitab4.5_0008522	16,455	19,128	4	2674	939	312	34,902.36	4.2426
Nitab4.5_0000259g0100	Nitab4.5_0000259	197,255	204,714	19	7460	2397	798	91,009.56	4.6568
Nitab4.5_0006030g0020	Nitab4.5_0006030	1168	4187	4	3020	1005	334	37,464.28	4.3656
Nitab4.5_0010244g0030	Nitab4.5_0010244	7441	12,807	20	5367	2376	791	89,985.44	5.0786
Nitab4.5_0012489g0020	Nitab4.5_0012489	27,033	29,689	4	2657	939	312	34,902.45	4.2361
Nitab4.5_0005256g0020	Nitab4.5_0005256	191,313	195,338	5	4026	897	298	33,176.67	4.4442

### Phylogenetic analysis of HSP90 gene families

**Fig. 2:**
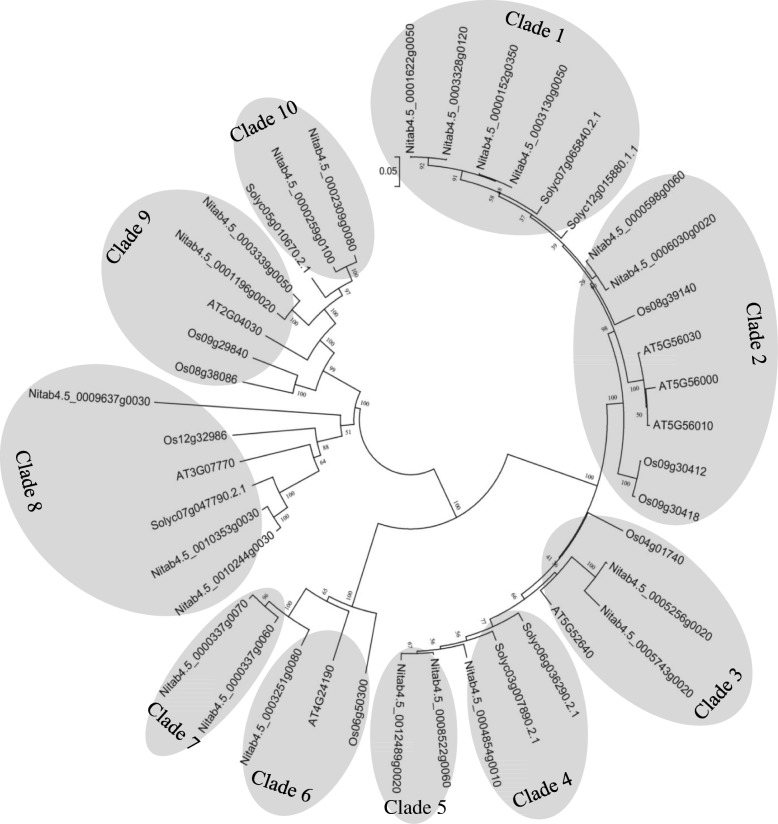
Phylogenetic analysis of HSP90 proteins from *Arabidopsis, Oryza sativa*, *Solanum lycopersicum,* and *Nicotiana tabacum*. The phylogenetic tree was constructed using MEGA6.0 software by neighbor-joining method with 1000 bootstrap replicates

In order to further elucidate the evolutionary relationship of HSP90 gene families, an unrooted phylogenetic tree was constructed using the neighbor-joining method with the MEGA6.0 software, including seven HSP90s from *Arabidopsis thaliana*, eight from rice, six from tomato, and twenty-one from tobacco. The phylogenetic tree branch of *Arabidopsis thaliana* was consistent with the previous studies [19]; the seven members could be divided into five subfamilies. As shown in Fig. 2, the phylogenetic tree indicated that the HSP90s were clustered into ten clades (Clade 1 to 10). Notably, Clade 8 contained the HSP90s from four species (*Arabidopsis thaliana*, rice, tomato, and tobacco). The clade with the largest number of HSP90 genes was Clade 2, which were 2 from tobacco, 3 from rice, and 3 from *Arabidopsis thaliana*. In addition, 24 HSP90 genes were found to be homologous, accounting for 57.14% (24/42) of the total number of HSP90 genes. There were 12 pairs of paralogs within the species, one of which was from *Arabidopsis thaliana* (AT5G56000 and AT5G56010), two pairs from rice (Os09g30412 and Os09g30418, Os09g29840 and Os08g38086), and nine pairs from tobacco.

### Exon-intron structure and phylogenetic analysis of the tobacco HSP90 gene family

**Fig. 3:**
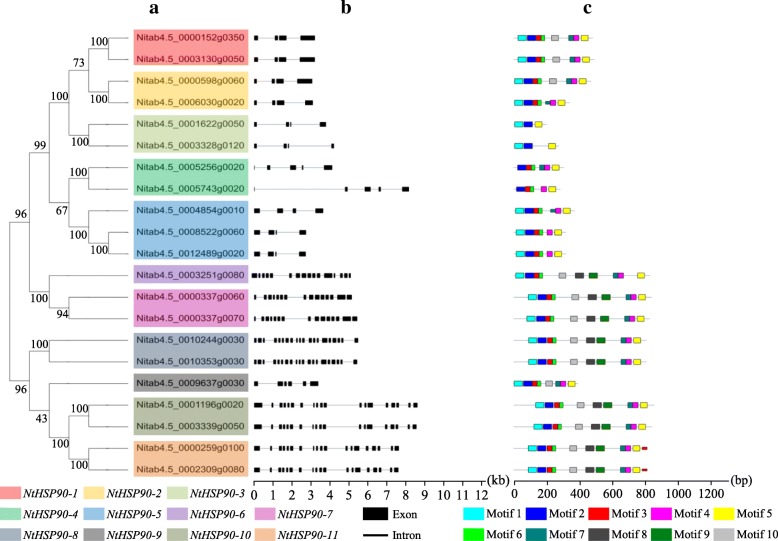
Phylogenetic tree, exon-intron structure, and motif analysis of *HSP90s*. **a** Phylogenetic analysis of NtHSP90 proteins in tobacco. **b** Gene structure analysis of NtHSP90s. Exons and introns are indicated in black rectangles and black lines, respectively. **c** Conserved motifs analysis of NtHSP90 proteins using MEME tools. Conserved motifs are showed in different colored boxes

Analysis of exon-intron structure can provide important insights into the evolution of gene families [39]. To analyze the exon-intron structure within the coding sequence in *NtHSP90s*, the genome and coding sequences of *NtHSP90s* were aligned using the Gene Structure Display Server (GSDS). A neighbor-joining phylogenetic tree was also constructed to explore whether the exon-intron distribution patterns correlate with the phylogenetic classification. The results showed that the HSP90 genes of tobacco could be clearly divided into eleven categories (*NtHSP90–1* to *NtHSP90–11*; Fig. 3a). As shown in Fig. 3b, we found 11 different exon-intron distribution patterns, showing a high degree of similarity in the same branch. This conservation of exon-intron structure patterns in each class strongly supported the close evolutionary relationships of *NtHSP90s* in the tobacco HSP90 gene family; all *NtHSP90s* contained introns in the genomic sequences. The number of introns of the HSP90 genes varied greatly in tobacco. The HSP90 gene of *NtHSP90–8* in the phylogenetic tree contained 19 introns, indicating that the gene was relatively complex. The HSP90 genes of *NtHSP90–1*, *NtHSP90–2*, *NtHSP90–3*, and *NtHSP90–5* only contained 3 introns (Fig. 3b). The *NtHSP90s* usually varied in exon-intron distribution patterns and gene lengths in different clades.

### Multiple sequence alignment and C-terminal conserved motifs analysis

We analyzed all of the conserved motifs of NtHSP90s and identified the pattern of amino acid residues conservation in their domains. It was showed that NtHSP90s contained 10 conserved motifs, containing 21 to 50 amino acids (Table 2, Fig. 3c). Among them, motif 6 contained the least number of amino acids (21). Four motifs (motif 1, 2, 8, and 9) contained 50 amino acids. The motif 2 was found in all NtHSP90 members.

**Table 2:** Conserved motif composition of the NtHSP90 proteins

Motif	Width	Multilevel consensus sequence
1	50	ETFAFQAEINQLLDLIINSFYSNKEIFLRELISNASDALDKIRFESLTDK
2	50	PELFIRIKPDKDNKTLTIIDSGIGMTKADLVNNLGTIARSGTKEFMEALQ
3	29	DVSMIGQFGVGFYSAYLVAEKVVVTTKHN
4	32	RIMKAQTLRDSSMSEYMRSKKYLEINPDHPIM
5	41	SVKDLVLLLFETALLTSGFSLDDPNTFGNRIYRMLKLGLSI
6	21	DDEQYVWESQAGGSFTVTRDV
7	29	VAKVQVSNRLSDSPCVLVTGKYGWSANME
8	50	ELFPRYLSFVKGLVDSDDLPLNVSREILQESRIVRIMKKRLVRKAFDMIQ
9	50	KFWENFGKFLKLGCIEDTGNHKRLAPLLRFFSSKSDEELISLDDYVENMK
10	41	KYWDWELTNETKPIWLRNPKEVEKEEYLEFYKKTFNEFLDP

**Fig. 4:**
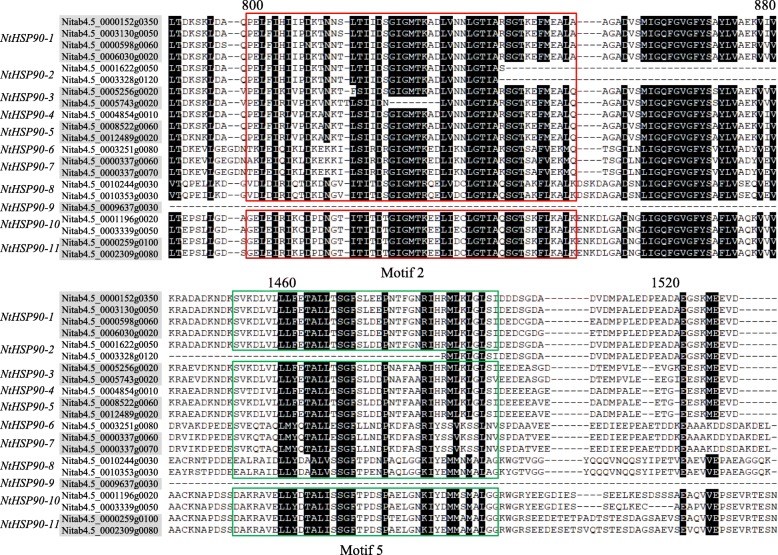
Multiple sequence alignment of the HSP90 domains in tobacco. The ATPase domain and signal peptide region are indicated by a red and green box, respectively

We performed a conserved motif analysis of NtHSP90s to obtain the pattern of amino acid residue conservation in NtHSP90 domains (Fig. 4). The results showed that the N-terminal domain was highly conserved, containing an ATPase site (red box). The conservative motif 2 was most widely distributed, which was closely related to the function of NtHSP90 N-terminal domain. Motif 2 constituted the ATPase domain of the NtHSP90 proteins, which functions as an ATP/ADP binding site with ATPase activity. Motif 5 was a low complexity sequence and had the function of signal peptide. Other motifs ensure the integrity of the NtHSP90 structures. Information of each motif, including motif logo and site number, was shown in Additional file 1: Figure S1.

### Expression patterns analysis

**Fig. 5:**
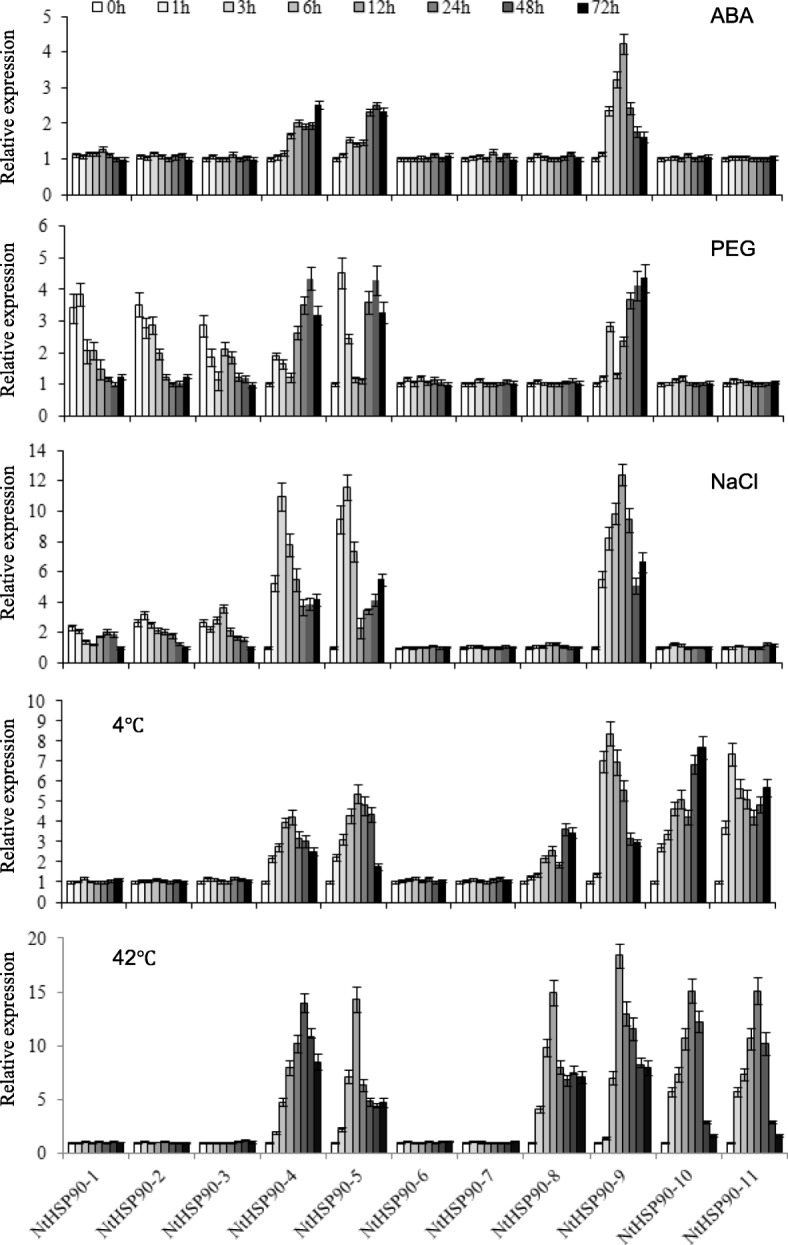
Expression patterns of *NtHSP90s* in response to ABA, PEG, NaCl, low and high temperature treatments. Transcript levels of *NtHSP90*s were analyzed by quantitative real-time PCR using *L25* gene as an internal control. The unstressed expression level (0 h) was regarded as a standard because of its lowest expression. Values are the mean ± SE, *n* = 3

Plants have formed a set of mechanisms for their own defense against stresses during long-term evolution. HSP90 genes are expressed as a response to abiotic stress [15]. To understand the expression patterns of *NtHSP9*0s under abiotic stress, the transcript level of 11 *NtHSP90s* subclasses was analyzed by qRT-PCR. As shown in Fig. 5, different expression patterns of different *NtHSP90s* were observed under ABA, drought, salt, cold and heat stresses. The expression of *NtHSP90–4*, *NtHSP90–5*, and *NtHSP90–9* were up-regulated, while *NtHSP90–6*, and *NtHSP90–7* were not induced by the above-mentioned five treatments. ABA treatment induced the weakest stress response among the five treatments. Although ABA treatment induced the transcriptions of *NtHSP90–4*, *NtHSP90–5*, and *NtHSP90–9* subclasses, the other 8 subclasses were not activated.

In response to drought (PEG treatment simulation) and NaCl treatments, there was a similar response pattern of *NtHSP90s* under osmotic stress. The transcriptions of *NtHSP90–1*, *NtHSP90–2* and *NtHSP90–3* were inhibited, whereas the transcript level of *NtHSP90–4*, *NtHSP90–5*, and *NtHSP90–9* were increased. The remaining genes were not induced by drought and salt stress. The expression patterns of different members after PEG treatment were variable and the times of peak expression were also not consistent. For the *NtHSP90–4*, *NtHSP90–5*, and *NtHSP90–9*, the expression levels induced by NaCl treatment were stronger than those induced by PEG treatment.

There was a similar expression pattern of *NtHSP90s* in response to cold and heat stresses. Six subclasses of *NtHSP90*s (*NtHSP90–4*, *NtHSP90–5*, *NtHSP90–8*, *NtHSP90–9*, *NtHSP90–10*, and *NtHSP90–11*) were notably up-regulated under cold and heat stresses. Generally, high temperature treatment showed the strongest stress response among the five treatments. Under cold stress, *NtHSP90–4*, *NtHSP90–5*, and *NtHSP90–9* showed high levels of transcription at 6–12 h; there was another peak at 48–72 h in expression levels of *NtHSP90–8*, *NtHSP90–10*, and *NtHSP90–11*. The expression patterns of six *NtHSP90*s under heat stress had unique expression profiles, responding with a single peak pattern showing high expression levels at 6–24 h.

## Discussion

Understanding the response of plants to high temperature stress is very important for plant growth [40]. Therefore, it is necessary to identify genes that are involved in heat shock responses in plants. High temperature stress usually changes the expression of related genes in the organism. When the temperature is (5 °C) higher than the normal temperature, most of the normal protein synthesis and mRNA transcription in the organism is inhibited. However, at the same time, a class of highly conserved proteins called HSPs is quickly synthesized. HSPs were first identified in the salivary gland chromosomes of Drosophila larva [41]. Later, other studies found organisms produce a series of proteins of different sizes, known as HSPs, in response to increased temperatures [42]. In addition to high temperature stress, abiotic stresses such as drought, salinity, heavy metals, and ABA could also induce the production of HSPs in plants [43]. HSPs have been classified into HSP100, HSP90, HSP70, HSP60, and sHSP according to their approximate molecular weights [15]. Among them, HSP90 is an important and highly conserved HSP; it is a molecular chaperone widely found in eukaryotic cells [44]. HSP90 genes have been reported to be involved in kinase and transcription factor folding, stress signal transduction, and DNA repair [29, 45, 46]. They play an important role in maintaining and regulating the conformation and function of intracellular proteins. The HSP90 has been identified in many plant species. However, there is little information about HSP90 in tobacco. Here, we focused on the correlation analysis of the tobacco HSP90 genes. The comprehensive identification and characterization of the HSP90 gene family in tobacco was facilitated by the recent completion of tobacco genome sequencing. The identification and analysis of the tobacco HSP90 gene family will provide valuable insights into the genetic improvement of other plants.

In the present study, 21 HSP90 genes were isolated and identified from the common tobacco database using bioinformatics methods (Table 1). Nine genes were located on chromosomes (chromosomes 1, 2, 5, 8, 9, 12, 18, and 23). Compared with other comprehensive surveys of plant HSP90 gene family (7 HSP90 family genes have been identified in *Arabidopsis*, 9 in rice) [19, 36], the tobacco HSP90 gene family is the largest with 21 phylogenetic extension genes. It may be related to the fact that common tobacco is an allotetraploid plant [47], whose HSP90 genes have been replicated. These HSP90 family proteins play a key role in the physiological maintenance and environmental adaptability of tobacco, enabling it to survive in high temperature stress and other stressful environments. The different HSP90 family proteins have different biophysical properties, which further indicate that there is wide diversity among members, which will help to further study the function of HSP90 genes. In this study, the isoelectric points of tobacco HSP90 ranged from 3.8808 to 5.9430. Moreover, all tobacco HSP90 proteins were acidic, which was consistent with the results for *Arabidopsis thaliana*, tomato, and others [48]. The tobacco HSP90 genes were non-homogeneously distributed on chromosomes, mainly on both ends of the chromosome (Fig. 1), which was similar to the distribution of rice HSP90 genes [36]. Gene duplication is an important mechanism in the evolution of gene families [49]. There were at least 6 pairs of repeated genes identified in tobacco, indicating that gene replication may occur during the evolution of the tobacco HSP90 genes.

Phylogenetic analysis is usually used to obtain insight into the evolutionary relationships of species and to help identify orthologs between species and paralogs within species. In this study, an unrooted phylogenetic tree was constructed based on the full-length protein sequences of *Arabidopsis*, rice, tomato, and tobacco. According to phylogenetic analysis, HSP90s could be divided into ten clades (Fig. 2). The orthologous genes from *Arabidopsis*, rice, tomato, and tobacco were clustered in the same branch (Clade 8), indicating that *NtHSP90s* were primeval than the divergence of dicots and monocots. There were 12 pairs of paralogs within species, implying that most species expanded according to their own species-specific approach during the evolution of the HSP90 gene family. This finding was consistent with the findings for gene families of cereals such as rice [50, 51].

The structure of protein determines its function [52]. The amino acid sequence of HSP90 family proteins can provide phylogenetic relationship information based on its primary structure. We found that there were different numbers of introns (3 to 19 introns) in different *NtHSP90* gene sequences. The number of introns is usually related to the sensitivity of gene transcription regulation. The lesser the number of introns, the stronger the plant’s ability to adapt to the diverse developmental processes and environmental stimuli [53]. The number of introns in *NtHSP90s* is the result of long-term evolution. According to the conserved motif analysis of the tobacco HSP90 genes, Nitab4.5_0001622g0050 and Nitab4.5_0003328g0120 contained fewer motifs, implying that it may have lost part of its sequence during evolution. Furthermore, there were 9 *NtHSP90s* that contained all 10 motifs, and their amino acid sequences were highly conserved. Motif 2 constituted the ATPase domain of the NtHSP90 proteins, which functions as an ATP/ADP binding site with ATPase activity [54]. The other nine motifs made up the tobacco HSP90 conserved domains, which play important roles in maintaining the complete ATPase domain activity [55].

Numerous studies have shown that the HSP90 genes are involved in response to abiotic stress [23, 56]. In the present study, we determined the dynamic expression levels of the *NtHSP90* genes under ABA, drought, salt, cold and heat stresses. The results showed that the expression of *NtHSP90–4*, *NtHSP90–5*, and *NtHSP90–9* were up-regulated, while *NtHSP90–6*, and *NtHSP90–7* were not induced by the above-mentioned five treatments. Therefore, we speculate that *NtHSP90–4*, *NtHSP90–5*, and *NtHSP90–9* are widely involved in the response to abiotic stresses, while *NtHSP90–6*, and *NtHSP90–7* may not be involved in regulation of abiotic stress tolerance in tobacco. These results showed that individual *NtHSP90* genes in the same clade may have distinct regulatory properties. High temperature treatment showed the strongest stress response among the different treatments, indicating that the *NtHSP90* genes were more sensitive to high temperature stress response. The expression of *NtHSP90s* was induced by ABA, drought, salt, cold and heat stresses, which may reflect their potential roles in abiotic stress response.

## Conclusions

In the present study, we systematically performed genome-wide identification and expression analysis of the tobacco HSP90 gene family, including gene structures, evolutionary relationships, chromosomal locations, conserved domains, and expression patterns. Twenty-one *NtHSP90s* were identified and classified into eleven categories. At least 6 pairs of *NtHSP90* genes underwent gene duplication, which arose from segment duplication and tandem duplication events. Expression pattern analysis indicated that *NtHSP90–4*, *NtHSP90–5*, and *NtHSP90–9* were induced by various abiotic stresses. *NtHSP90s* were strongly induced by heat stress, while weakly activated by ABA treatment. There was a similar response pattern of *NtHSP90s* under osmotic stress, or extreme temperature stress. The results provide a basis for further study of the biological functions of Hsp90 genes in response to abiotic stress.

## Methods

### Plant materials and stress treatments

The tobacco cultivar K326 (*Nicotiana tabacum* L., cv. Kentucky 326) was used for gene expression level related experiments. The tobacco seeds were sown in mixed soil (vermiculite:humus = 1:1) saturated with water in sieve-like plates. Seedlings were germinated and cultured in a greenhouse at 22 °C with a 16 h/8 h (light/dark) photoperiod for eight weeks. Then, they were assigned to a treatment group and separately stressed by exposure to a 50 μM ABA spray, a PEG 6000 solution (− 0.5 MPa), a 300 mM NaCl solution, a low temperature (4 °C), and a high temperature (42 °C), all of which were validated to cause a significant stress in pilot experiments. Untreated control plants were cultured normally. All the true leaves were sampled at 0, 1, 3, 6, 12, 24, 48, and 72 h after treatment. The main midribs were then removed. After collection, all samples were quickly frozen in liquid nitrogen and stored at − 80 °C for RNA isolation and analysis.

### Identification of *NtHSP90* genes

The *Arabidopsis thaliana* HSP90 proteome sequences were downloaded from the TAIR databases (http://www.arabidopsis.org/) [19]. The protein sequences of *Arabidopsis thaliana* HSP90 genes were used as query to perform BLASTP (E-value 1e-10) searched against *N. tabacum* genome sequences to obtain the final dataset of *NtHSP90s* (https://solgenomics.net/). Redundant sequences were removed. Then the Pfam (http://pfam.sanger.ac.uk/search) and SMART (http://smart.embl-heidelberg.de/) databases were used to confirm each predicted HSP90 protein [57, 58]. The sequences of rice HSP90s and tomato HSP90s were obtained from the Rice Genome Annotation Project (http://rice.plantbiology.msu.edu/) and the Solanaceae Genome Database (https://solgenomics.net/). The biophysical properties of coding HSP90s were calculated using the Expasy ProtParam tool (http://us.expasy.org/tools/protparam.html) [59].

### Phylogenetic analysis

Multiple sequence alignment was carried out by using MUSCLE based on the sequences of *Arabidopsis thaliana*, rice, tomato, and tobacco [60]. The MEGA 6.0 software was used to construct an unrooted phylogenetic tree using the neighbor-joining method [61]. Support for the tree topology was evaluated by using a bootstrap analysis with 1000 replicates.

### Gene structure analysis

A diagrammatic sketch of the HSP90 gene structure was constructed using the Gene Structure Display Server (GSDS) (http://gsds.cbi.pku.edu.cn/) [62]; it was based on the alignment of the cDNAs with their corresponding genomic DNA sequences.

### Multiple sequence alignment and motif analysis

Alignment of multiple HSP90 protein sequences from tobacco was performed using ClustalW (http://www.genome.jp/tools/clustalw/) [63]. The parameters were set to default values and the results of the alignment were visualized using the BoxShade program. The conserved motif of the full length of HSP90 family proteins was analyzed using the online MEME tool (Multiple Expectation Maximization for Motif Elicitation, http://meme-suite.org/tools/meme) [64]. The maximum motif search value was set at 10.

### Chromosome distribution and synteny analysis

The distribution information of NtHSP90 gene family in the chromosome was obtained from the Sol Genomics Network (https://solgenomics.net/) database. For synteny analysis, synteny blocks containing HSP90 genes in the tobacco genome were identified using MCScanX program [65]. The chromosome distribution for each *NtHSP90* and synteny relationship were displayed with circos (http://circos.ca/) [66].

### Quantitative RT-PCR expression analysis of *NtHSP90s*

Total RNA was extracted from the samples using a modified CTAB method [67]. After removing genomic DNA contamination by DNase I (Fermentas, Waltham, MA, USA), 1 μg of total RNA was reverse-transcribed to cDNA using the PrimeScript™ RT Reagent Kit (Takara, Dalian, China). The PCR amplifications were performed using LightCycler® 480II (Roche Diagnostics, Indianapolis, IN, USA). For qRT-PCR, gene-specific primers were designed according to the cDNA sequences using Primer 6.0. Details of primers are shown in Additional file 2: Table S1. The transcription of tobacco ribosomal protein gene *L25* (GenBank accession number L18908) was used as an internal reference gene. The qRT-PCR reactions were performed on the ABI 7900 HT Real-Time PCR System (Applied Biosystems) using the following thermal cycle: 95 °C for 5 min, followed by 40 cycles at 95 °C for 10 s, and 60 °C for 30 s. Three biological replicates were used for each gene. The relative expression level for each of *NtHSP90s* was calculated using the 2^–ΔΔCT^ method [68].

## Additional files

Additional file 1: **Figure S1.** Motif analysis of the NtHSP90 proteins. The 10 motifs were analyzed using the MEME online tool. Different letters represent the abbreviation of various amino acids. The higher the letter height, the stronger the conservatism of the amino acid at that position. (PPTX 417 kb)

Additional file 2: **Table S1.** Specific primers of *NtHSP90* in qRT-PCR. (DOCX 16 kb)

## Abbreviations

ABA: Abscisic acid
HSP90: Heat shock protein 90
*NtHSP90*: Gene in *Nicotiana tabacum*
PEG: Polyethylene glycol
pI: Isoelectric point
qRT-PCR: Quantitative real-time PCR
