# Supplementary figures and images for: Genome-wide identification and expression analysis of HSP90 gene family in Nicotiana tabacum
Source: BMC Genet. 2019 Mar 19;20:35. doi: 10.1186/s12863-019-0738-8 (PMC6423791; doi:10.1186/s12863-019-0738-8)

## Slide 1
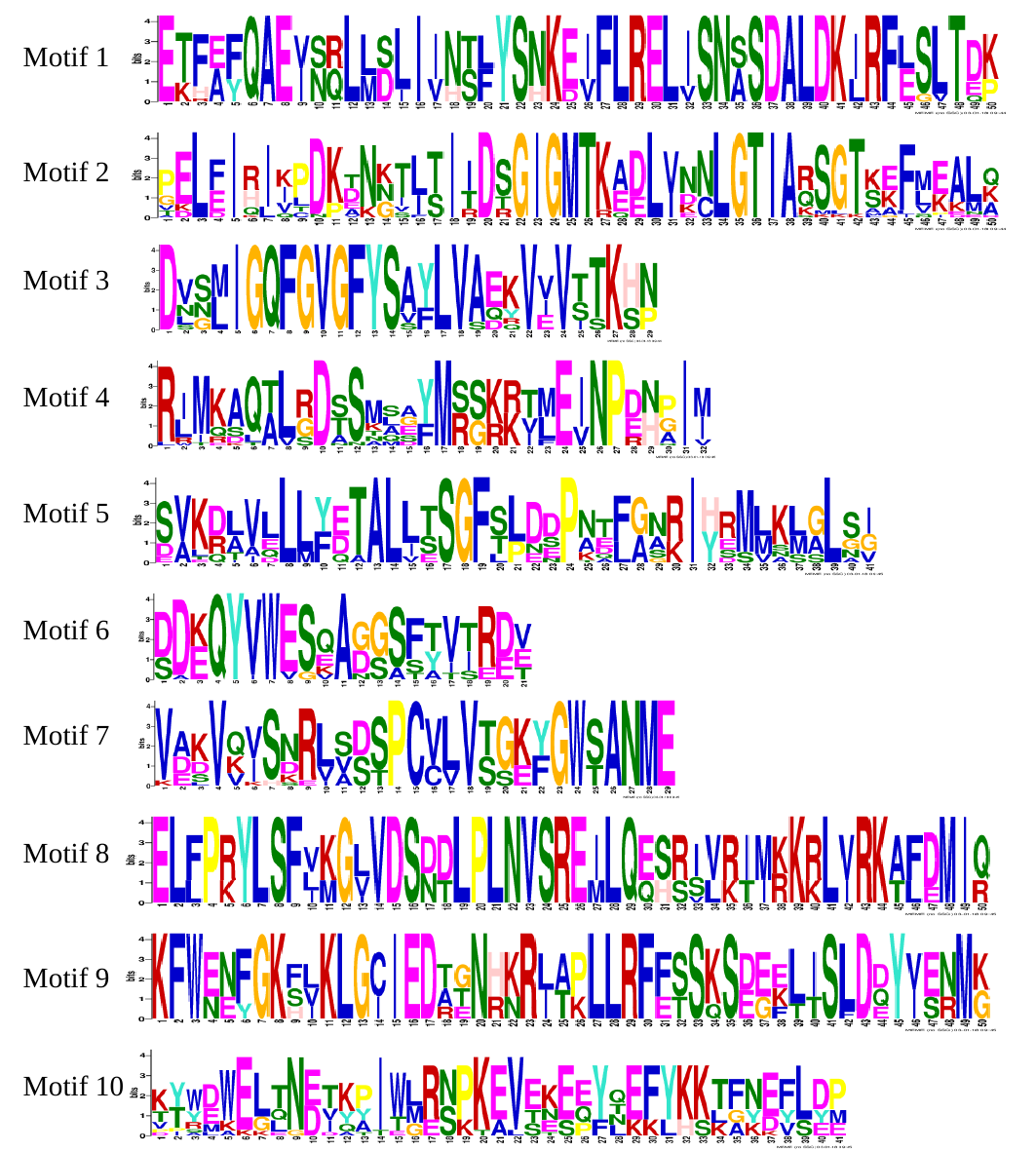

Motif 1
Motif 2
Motif 3
Motif 4
Motif 5
Motif 6
Motif 7
Motif 8
Motif 9
Motif 10

Supplement: Supplementary file 1 — Figure S1. Motif analysis of the NtHSP90 proteins. The 10 motifs were analyzed using the MEME online tool. Different letters represent the abbreviation of various amino acids. The higher the letter height, the stronger the conservatism of the amino acid at that position. (PPTX 417 kb) [file 12863_2019_738_MOESM1_ESM.pptx]
