# Supplementary material for: Genome-wide identification and expression analysis of HSP90 gene family in Nicotiana tabacum
Source: BMC Genet. 2019 Mar 19;20:35. doi: 10.1186/s12863-019-0738-8 (PMC6423791; doi:10.1186/s12863-019-0738-8)
Supplement: Supplementary file 2 — Table S1. Specific primers of NtHSP90 in qRT-PCR. (DOCX 16 kb) [file 12863_2019_738_MOESM2_ESM.docx]

**Table S1** Specific primers of *NtHSP90* in qRT-PCR

| Primer name | Forward primer (5´→ 3´) | Reverse primer (5´→ 3´) |
| --- | --- | --- |
| *NtHSP90-1* | ACCACAAAGCACAATGATGAT | CCTTCAAGAGAGAGATTGGGTA |
| *NtHSP90-2* | ATGGCTGATGCTGAAACCT | GGAACAAGGCGAATGAAGA |
| *NtHSP90-3* | TGGCGGACACAGAGACCT | TGGTCTTGTCAGGAATAATGTG |
| *NtHSP90-4* | TTGGATAAGATTCGGTTTGA | TCGTCACCACAACTTTCTCA |
| *NtHSP90-5* | TTTTCTCAATGGCGGATG | TGGAGAGAGTCTTGTTAGCCT |
| *NtHSP90-6* | TCGTTCACTGTTACAAGGGA | AATGCTTTTTCACAAGGTCC |
| *NtHSP90-7* | TTGTCCCTCCTAAGGCTCC | CTCTTGAGACATTGAGGGGTA |
| *NtHSP90-8* | AAGTTGAGGTTTCTTGGTGTTA | GAGTAGAATCCCACGCCG |
| *NtHSP90-9* | AGGAAGGAGAAGATGCGAC | AGAAGCAAGAGGCTCCAAA |
| *NtHSP90-10* | CAAAGAGCCCAAGGTCAGA | TCCTCCTCTACCTCAATTGTT |
| *NtHSP90-11* | GTTGACATAAGCAAGGAGG | TTCCAGATACAAGCACGCA |
